# Intracardiac mass in a 3-month-old boy revealing a congenital syphilis

**DOI:** 10.1016/j.bjid.2026.104613

**Published:** 2026-01-20

**Authors:** Edouard Martinez Casado, Ludovic Lemée, Elise Barre, Didier Pinquier, Adnan Hassani, Hortense Petat

**Affiliations:** a Rouen University Hospital, Pediatric Department, Rouen, France; b University Hospital of Rouen, Department of Microbiology, Rouen, France; c Rouen University Hospital, Department of Neonatology, Pediatric Intensive Care, Rouen, France; d Centre Hospitalier Universitaire de Rouen, Pediatric Radiology Department, Rouen, France; e Univ Rouen Normandie, Université de Caen Normandie, INSERM, Normandie Univ, Rouen, France

**Keywords:** Congenital syphilis, Heart gumma, Syphilis

## Abstract

The rate of congenital syphilis remains low in developed countries, as most women with syphilis are routinely screened and treated during pregnancy. We report here the case of a 3-month-old boy, with fever that lasted 15-days referred to the emergency department of tertiary care paediatric center. He had no history and had never travelled. Examination showed severe pallor, slight forehead bulging and large isolated hepatomegaly. Blood tests found: anaemia, elevated C-reactive protein and hyperlymphocytosis. Echocardiography found an intracardiac mass on the posterior wall of the right atrium extending to the origin of the superior vena cava. Cardiac MRI confirmed that mass. The Treponemal Hemagglutination Assay (TPHA) and non-treponemal test (Venereal Disease Research Laboratory, VDRL) were highly positive. Prolonged antibiotic therapy with penicillin G resulted in good clinical evolution, disappearance of lymphocytosis and total disappearance of the mass at 20-months' follow-up.

## Background

Worldwide, over 5-million new cases of syphilis are diagnosed each year, with most infections occurring in low- and middle-income countries, where infections are endemic and congenital infections are not uncommon.^1^^,^^2^ Thanks to systematic mandatory and free screening of all pregnant women in France, the rate of congenital syphilis remains low (< 1/100,000 live births), as most women with syphilis are treated during pregnancy. Penicillin treatment of pregnant women with syphilis is 98 % effective in preventing congenital syphilis.^3^ Otherwise, since the year 2000, an increase of syphilis cases is noted in France. Between 2013 and 2015, there was an increase in female (+85 %) and male (+75 %) cases through the heterosexual population. Low socio-economic level and precariousness are risk-factors. This increasing rate of primary or secondary syphilis was also observed in US women between 2011 to 2016 and consequently an increasing rate of mother-to-child transmission. We report here the rare case of a neonatal syphilis with a cardiac involvement.

## Case report

A 3-month-old boy was admitted with a fever that lasted for 15-days. He was born at full term, and had no antecedents or risk factors for tuberculosis, and had not travelled since birth. The mother had been monitored during pregnancy, and no risk factors had been identified; the recommended serologies were performed and early screening was negative for HIV and syphilis and revealed previous immunization for rubella and toxoplasmosis. On admission to the emergency department, a complete physical examination showed: severe pallor, slight forehead bulging and large isolated hepatomegaly. He had normal vital parameters, with a hetero-rated pain scale of 9/15.

**Fig. 1:**
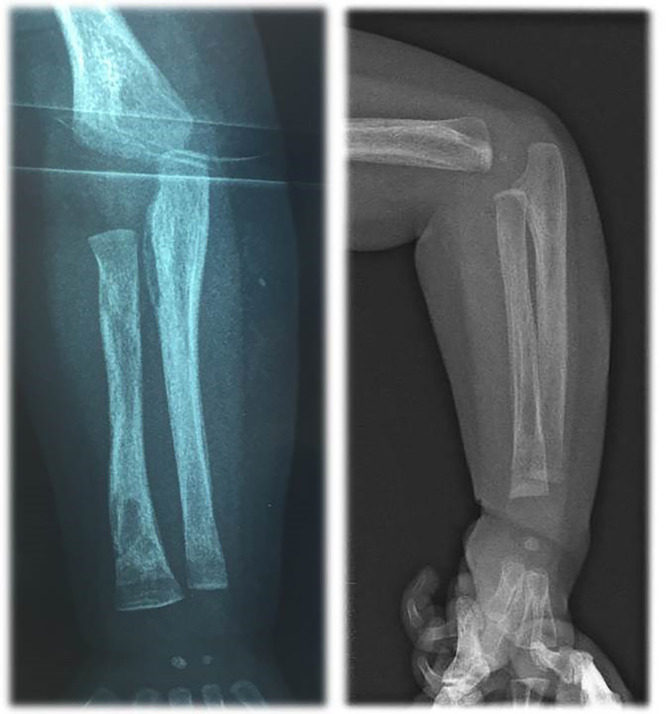
Lower part of radius periostitis and osteomyelitis before antibiotics (a) and after one month after antibiotics treatment (b).

**Fig. 2:**
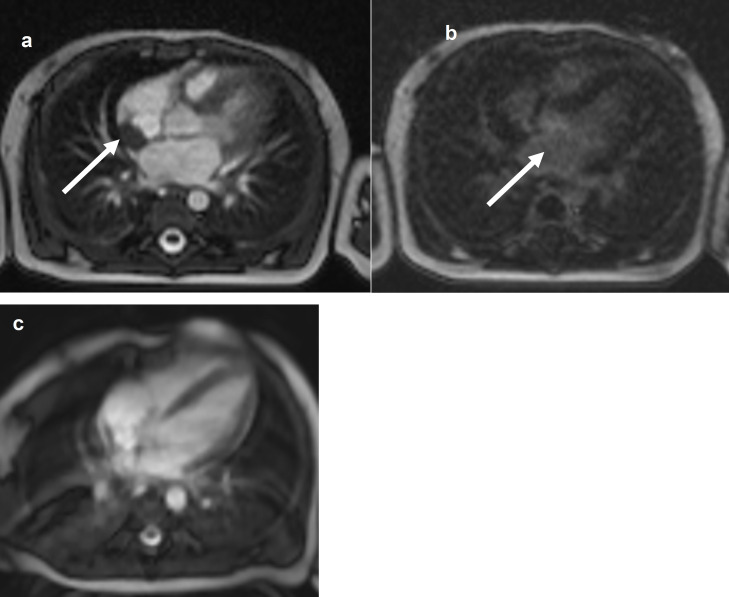
Cardiac-MRI comparison of intracardial mass (arrow) between the diagnosis and (a‒b) 20-months follow-up (c). T2 TrueFISP Sequence: Right intra-auricular mass at T2, attached to the posterior surface of the atrium and to the posterior part of the inter-atrial septum. Sequence after gadolinium injection: No lesion enhancement. Complete regression of intra-atrial mass.

Blood tests showed: aregenerative normocytic anaemia (7.7 g/dL of hemoglobin), elevated C-reactive protein (160 mg/L), elevated white blood cells (28 G/L) with a high lymphocytosis (13 G/L). Liver tests showed mild cytolysis without cholestasis. Chest X-Ray was normal. Urine cytology and culture, lumbar fluid tests and culture, repeated blood cultures, and viral serologies were negative. Empirical antibiotic treatment with amoxicillin/clavulanate was initiated, then replaced by cefotaxime for six days without improvement. Because of the persistence of fever despite antibiotic treatment, other infectious causes were investigated: a ^99m^Tc-diphosphonates scintigraphy showed a left wrist hyperfixation, and the further wrist X-Ray diagnosed a periostitis and osteomyelitis of the radius (Fig. 1), but the lesions were too small for biopsy. The antibiotics were then replaced by cefazolin and clindamycin for six days. MRI of the left wrist did not confirm osteomyelitis; so, antibiotics were discontinued. Echocardiography showed an intracardiac mass (15 × 8 mm) on the posterior wall of the right atrium extending to the origin of the superior vena cava. Cardiac MRI showed an intracardiac mass attached to the posterior wall and interatrial septum with a T2 hypo-signal, without enhancement after gadolinium injection (Fig. 2). Three hypotheses were considered: a right atrial myxoma (unlikely, however, due to non-enhancement after gadolinium injection), thrombosis of the superior vena cava, or a large Chiari network, which is a fenestrated, net-like embryonic remnant of the sinus venosus valves. One month later, in view of the finding of multifocal involvement, and persistent fever with persistent lymphocytosis elevation of liver enzymes, toxoplasmosis and syphilitic serology were performed. The Treponemal Hemagglutination Assay (TPHA) was superior to 10,240 and the VDRL (non-treponemal agglutination assay) was equal to 1:32. A new test on the mother revealed positive VDRL (1:8) and TPHA (> 10,240) tests. This child was born into a family with a low economic and social status, and the mother later reported several sexual partners. The infant was treated for 10-days with penicillin-G, administered at a dose of 250,000 units/kg/day, as recommended.^4^ Fever subsided on the first day, but a study of lymphocyte count and CRP showed that the inflammation had not been resolved. Thirteen days after the end of antibiotics, a second antibiotic course was prescribed: penicillin-G, administered at a dose of 150,000 units/kg/day for 15-days. Then a third cure was prescribed 10-days after the end of the second one, with the same posology. The effect of this prolonged antibiotic therapy led to a negative CRP and good infant health. However, the right atrial mass did not decrease and hyperlymphocytosis persisted. The infant was discharged after 4-months in hospital.

**Fig. 3:**
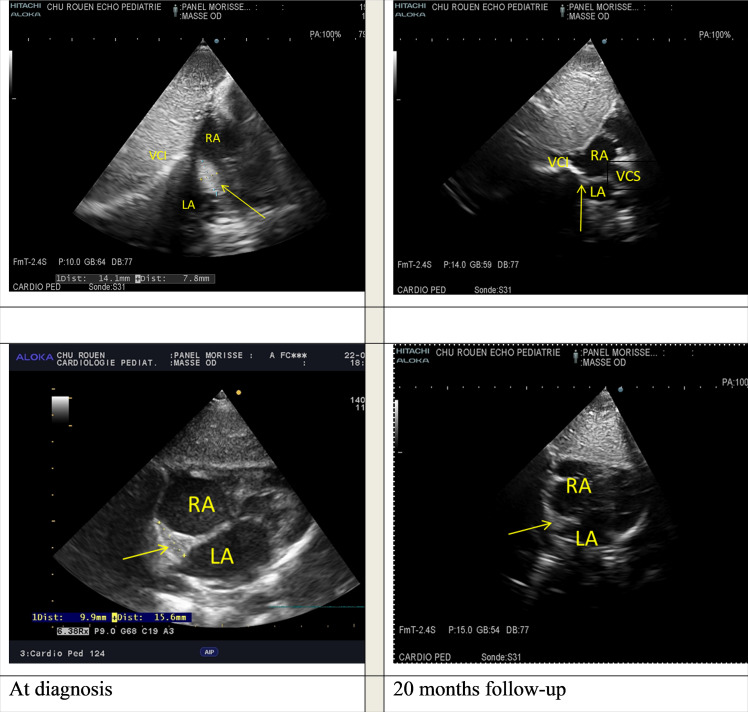
Echocardiographic views comparison of intracardial mass (yellow arrow) between the diagnosis and 20-months follow-up. RA, Right Atrium; LA, Left Atrium; IVC, Inferior Vena Cava; SVC, Superior Vena Cava.

Follow-up at 24-months showed a good clinical evolution, with slow disappearance of the cardiac mass, with negativation of VDRL. TPHA rates remained positive and stable over the course of the evolution. The boy showed good psychomotor development and growth. No other signs of syphilis were found. The intracardiac mass disappeared at the 20-month follow-up echocardiography (Fig. 3Figure 3).

## Discussion

We report here the case of a presumed congenital syphilis with a rare heart improvement. Some infants with early congenital syphilis are asymptomatic at birth. Clinical manifestations of early congenital syphilis might be neonates rhinitis, hepatomegaly, jaundice, generalized lymphadenopathy, rash and osteochondritis. Late-onset congenital syphilis occurs when the diagnosis is unknown, and persistent inflammation can lead to clinical signs, such as: skin and mucous membrane scratching, perioral fissures, frontal bumps, saddle nose, prominent maxilla, saber shins, Hutchinson's teeth, intellectual disability and cranial nerve palsies, sensorineural hearing loss and vision changes, interstitial keratitis, secondary glaucoma and corneal scarring.

Transmission to the foetus generally occurs via the placenta. The risk of vertical transmission of syphilis by an infected, untreated mother decreases as maternal disease progresses, ranging from 70 %‒100 % for primary syphilis and 40 % for early latent syphilis to 10 % for late latent disease.^5^ Seroconversion during pregnancy is rare but potentially serious. Around 40 % of pregnancies in women with untreated early syphilis result in perinatal death.^6^^,^^7^ The most common cause of foetal death is placental infection associated with reduced blood flow to the foetus, which also explains why many live-born infants are premature or of low birth weight, although direct foetal infection also plays a role.^8^

In our case report, we were destabilized by the intracardiac mass. We did not know if it was a localization of syphilis like a “gumma”. Gummas usually occur after years or decades in untreated adult syphilis patients.^1^^,^^9^ A few exceptional and historical cases are described, but they do not concern infants.^10^, ^11^, ^12^, ^13^ Although we do not have a biopsy of the mass, we have come to this diagnosis on a cluster of arguments: high exposure to Treponema during pregnancy (seroconversion after first trimester of gestation), early contamination during pregnancy due to the impact on the foetus with low birth weight, mid-term follow-up showed mass decrease with decline of VDRL, and exclusion of differential diagnoses with cardiac MRI. In this case, the decrease in syphilis markers associated with the child's clinical improvement, as well as the normalization of the cardiac ultrasound, allowed us to confirm the diagnosis without performing a biopsy, which was too dangerous for the child.

With regard to treatment, we followed the 2015 Sexually Transmitted Diseases Treatment Guidelines for the treatment of infants with congenital syphilis aged ≥1-month.^8^ Although general health improved with treatment, the lack of consensus on management of intracardiac syphilis disorders at this age prompted us to perform two further courses of penicillin-G, believing that potential intracardiac “gumma” was more difficult to treat according to tertiary stage. Finally, the intracardiac mass did not immediately decrease, and disappeared 20-months without modification of TPHA level. Disappearance suggests that this was syphilitic heart “gumma”.

## Conclusion

This case reminds us that congenital syphilis is a disease with a polymorphous presentation. In infants, this diagnosis must be seriously considered in the face of an infectious presentation with multifocal involvement. Cardiac syphilitic “gumma” in congenital syphilis is a unique case. This observation highlighted the need to repeat Treponema testing in high-risk pregnancies at 6-months of gestation and at birth.

## Ethical approval

Not required.

## Funding

None.

## Data availability statement

The data that support the findings of this study are available from the corresponding author upon reasonable request.

## Conflicts of interest

The authors declare no conflicts of interest.
